# Progress in Biomaterials for Cardiac Tissue Engineering and Regeneration

**DOI:** 10.3390/polym15051177

**Published:** 2023-02-26

**Authors:** Alexandru Scafa Udriște, Adelina-Gabriela Niculescu, Luminița Iliuță, Teodor Bajeu, Adriana Georgescu, Alexandru Mihai Grumezescu, Elisabeta Bădilă

**Affiliations:** 1 Department 4 Cardio-Thoracic Pathology, “Carol Davila” University of Medicine and Pharmacy, 050474 Bucharest, Romania; 2 Research Institute of the University of Bucharest—ICUB, University of Bucharest, 050657 Bucharest, Romania; 3 Department of Science and Engineering of Oxide Materials and Nanomaterials, Politehnica University of Bucharest, 011061 Bucharest, Romania; 4 Pathophysiology and Pharmacology Department, Institute of Cellular Biology and Pathology “Nicolae Simionescu” of the Romanian Academy, 050568 Bucharest, Romania; 5 Academy of Romanian Scientists, Ilfov No. 3, 050044 Bucharest, Romania; 6 Cardiology Department, Colentina Clinical Hospital, 020125 Bucharest, Romania

**Keywords:** cardiac tissue engineering, cardiac regeneration, stem cells, biomaterials, cardiac patches, injectable hydrogels, scaffolds, extracellular vesicles

## Abstract

Cardiovascular diseases are one of the leading global causes of morbidity and mortality, posing considerable health and economic burden on patients and medical systems worldwide. This phenomenon is attributed to two main motives: poor regeneration capacity of adult cardiac tissues and insufficient therapeutic options. Thus, the context calls for upgrading treatments to deliver better outcomes. In this respect, recent research has approached the topic from an interdisciplinary perspective. Combining the advances encountered in chemistry, biology, material science, medicine, and nanotechnology, performant biomaterial-based structures have been created to carry different cells and bioactive molecules for repairing and restoring heart tissues. In this regard, this paper aims to present the advantages of biomaterial-based approaches for cardiac tissue engineering and regeneration, focusing on four main strategies: cardiac patches, injectable hydrogels, extracellular vesicles, and scaffolds and reviewing the most recent developments in these fields.

## 1. Introduction

Cardiovascular diseases (CVD) comprise a group of life-threatening pathological disorders that represent the leading cause of mortality worldwide. As the adult heart has a limited regeneration ability, cardiac injury leads to progressive function deterioration, resulting in heart failure [1,2,3,4]. Particularly, myocardial infarction (MI) is a frequent cause of heart failure as it induces irreversible cardiomyocyte loss, scar formation, altered myocardial architecture, thin and weakened ventricular walls, and arrhythmias [4,5,6].

**Figure 1:**
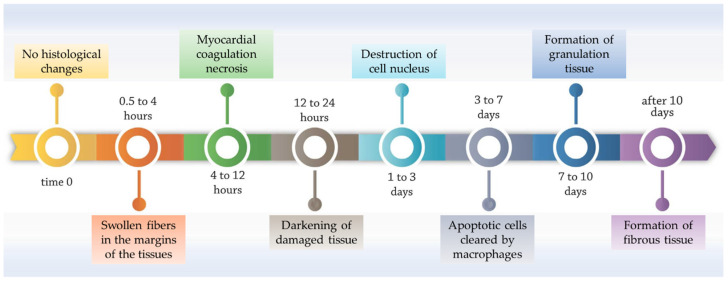
Histological changes after MI on heart tissue, including the period of 0–10 days. Adapted from [7].

Immediately after an MI, no histological change is visible under the microscope. However, within the period of 30 min–4 h, swollen fibers are observed in the margins of the affected tissues, and glycogen is lost. Within the next 8 h, myocardial coagulation necrosis occurs, leading to swelling of the area. During the 12–24 h period, the darkening of damaged tissue is noticed, with the accumulation of neutrophils. The cell nucleus is destroyed on days 1–3, while in the following period (i.e., days 3–7), macrophages clear apoptotic cells from the injured area. On days 7–10, the formation of granulation tissue occurs, after which type I collagen settles in the area. Finally, within 2 months, the formation of fibrous tissue is observed, thus replacing dead cells and scars [7,8]. For clarity, the timely evolution of histological changes post-MI is represented in Figure 1.

In the necrosis phase, apoptotic cardiomyocytes release reactive oxygen species (ROS) and other intracellular contents into the niche, triggering pro-inflammatory cytokines and the recruitment of immune cells into the injured area. The inflammatory microenvironment further contributes to homeostasis disruption and increases oxidative damage, aggravating inflammation and fibrosis. Thus, an orchestrated biological and mechanical therapeutic strategy is imposed to efficiently mediate pathological processes in the necrosis, inflammation, and fibrosis phases [9].

Unfortunately, beyond pharmacological therapy, the main treatment possibilities for post-MI heart failure remain heart transplantation and cell-based therapies [2,10,11]. Nonetheless, these cardiac therapies face several difficulties, including the small number of donors, the necessity of immunosuppressive drug administration following surgery, and the limited success of cell therapies due to challenging delivery, poor integration, and implanted cell survival [4,10,11,12].

In this context, better strategies must be sought to ensure effective and efficient cardiac regeneration. Therefore, intense research efforts have been directed to integrate bioengineering concepts in optimized cardiovascular treatments to overcome the shortcomings of current therapies [13,14]. One particularly appealing interdisciplinary approach that has gained ground in the past years is cardiac tissue engineering by combining various biomaterials, cells, and bioactive molecules for repairing and restoring heart tissue [4,10,15,16]. Specifically, various natural and synthetic biomaterials can act as both delivery platforms and support structures for cells to proliferate and differentiate into healthy cardiac tissue.

In this respect, recent studies have exploited the variety and versatility of biomaterials for creating innovative patches, hydrogels, scaffolds, and delivery platforms that hold tremendous promise for future clinical utility. Even though some of these topics have been addressed in previous reviews [11,14,17,18,19,20,21], this paper aims to provide an updated outlook on the subject. Hence, this review focuses on the most recent developments in biomaterials used for cardiac tissue engineering and regeneration, mostly discussing studies published between 2018 and 2022 while offering several future perspectives.

More specifically, this paper briefly discusses cell-based therapy limitations, emphasizing the need for biomaterial-based approaches. Then, the following sections aim to present in more detail the identified novelties in the field of cardiac tissue engineering and regeneration, focusing on recently developed cardiac patches, injectable hydrogels, extracellular vesicle-based therapies, and advanced scaffolds. Through this comprehensive pathway, this review aspires to present the progress in the field of biomaterials for cardiac repair, serve as an inception point for future research, and help envisage more efficient treatment strategies.

## 2. Cell-Based Therapies

**Figure 2:**
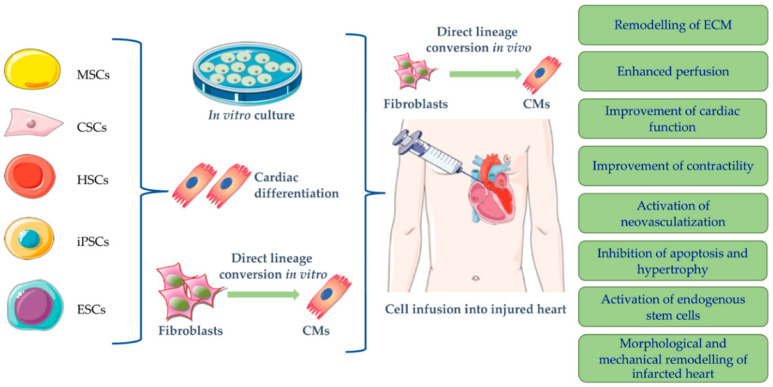
Overview of stem cells investigated for cardiac regenerative therapy. Adapted from [22]. Abbreviations: MSCs—mesenchymal stem cells; CSCs—cardiac stem cells; HSCs—hematopoietic stem cells; iPSCs—induced pluripotent stem cells; ESCs—embryonic stem cells; CMs—cardiomyocytes; ECM—extracellular matrix.

Cell therapy is one of the recently considered strategies for cardiac regeneration, consisting of direct injection of exogenous therapeutic cell suspension into the affected heart or activation of endogenous regenerative processes through stimulation of adult tissue-restricted stem cells [11,22]. In this respect, a broad range of stem cells at different developmental stages has been researched in relation to their ability to replace damaged or dead cardiomyocytes toward improving cardiac function and ensuring heart tissue regeneration. Cells ranging from adult stem or progenitor cells to embryonic or induced pluripotent stem cells have been taken into consideration for creating efficient treatments [4,23,24,25,26] (Figure 2). Specifically, scientists have investigated in vitro and in vivo testing of bone marrow-derived stem cells [27,28,29], cardiac stem cells [29,30], induced pluripotent stem cells [31], pluripotent stem cell-derived cardiomyocytes [32], pluripotent stem cell-derived mesenchymal stromal cells [33], adipose-derived stem cells [34,35], embryonic stem cells [36], fetal membrane-derived mesenchymal stem cells [37], menstrual blood-derived endometrial stem cells [38], and more.

In addition to their pluripotency and self-renewal capacity, stem cells are also endowed with paracrine effects, anti-inflammatory activity, and immunomodulatory capacity [24,26,39]. Moreover, the high cardiac differentiation potential and the possibility to develop large-scale cultivation systems render cell-based therapies promising for achieving great progress in unveiling MI’s molecular and cellular mechanisms [40,41].

Nonetheless, the efficacy of cell-based therapies is impeded by several drawbacks, counting low retention and engraftment of transplanted cells, the potential for differentiation into host cell types, viability under the harsh conditions of damaged tissue, and risk of inflammation and immunoreaction [1,7,24,42]. In addition, from the wide range of available cell candidates, only bone marrow-derived stem cells, myoblasts, cardiac progenitor cells, and adipose-derived stem cells have been involved in clinical trials, leading to mixed encouraging and disappointing results. However, negative outcomes might have been caused by the poor permanence of the injected cells inside the tissue [13]. In more detail, the environment of the post-MI heart is relatively acidic and presents a severely affected extracellular matrix (ECM), hampered mechanical properties causing a higher ventricular applied tension than that of the threshold of the defective tissue. Hence, the unfavorable environment causes a significant volume of cells to suffer apoptosis at a short time after transplantation, leading to overall unsatisfactory results [43]. To overcome these limitations, biomaterials appeared as a convenient solution. Seeding cells to a scaffolding material can improve cell retention and engraftment, enhancing cell survival and restoring cardiac function [1,39,42].

## 3. Biomaterial-Based Approaches

### 3.1. Biomaterials—Brief Overview

Biomaterials comprise a broad field of different scaled materials from macro- to micro- and nano-sized polymers, ceramics, metals, and composites that can work with living matter to replace or restore damaged tissues [12]. They can be used in tissue engineering in various forms (e.g., carriers, hydrogels, scaffolds), playing a vital role in anchoring cells and serving as a framework for their further proliferation and differentiation [44].

For the specific purpose of cardiac tissue engineering, biomaterial structure and function should mimic the native features of cardiac ECM and furnish a proper microenvironment for enhancing cell viability [1,5]. Specifically, biomaterials aiming to ensure cardiac repair should be biocompatible and biodegradable, exhibit similar mechanical and biological properties to native myocardium, enable cell integration, diminish the hostility of the local microenvironment, ensure a slow release of bioactive molecules, and provide appropriate electrical conductivity [1,12,45]. Moreover, integrating antioxidant agents within biomaterials has the potential to mediate inflammation and fibrosis. By scavenging ROS, antioxidant structures can attenuate oxidative damage, pro-inflammatory polarization of macrophages, and fibrotic response to cardiac repair [9].

Despite the complex requirements for a suitable biomaterial, various natural (e.g., fibrin, gelatin alginate, chitosan, collagen, hyaluronic acids, silk) and synthetic polymers (e.g., polyglycolic acid (PGA), polylactic acid (PLA), polylactic-co-glycolic acid (PLGA), polyurethane (PU) and their derivatives) have been reported to resemble (in various degrees) several ECM properties of interest [5,12,44,46]. Their beneficial features include biocompatibility, appropriate chemistry, hydrogel formation ability, suitable mechanical properties, and satisfactory degradation rate [5,15,47].

Further processing of convenient materials allows their tailoring with appropriate morphology and characteristics for application in cardiac tissue engineering [44]. Moreover, stem and/or progenitor cells of interest can be mixed with or cultured on biomaterials [12]. This results in a synergic combination as cells endow biomaterials with the capacity of tissue reconstruction and regeneration, while biomaterials offer the necessary support for cell-to-tissue processes, such as cell–cell adhesion, proliferation, and differentiation [44,47]. To improve restorative potential, biomaterials can also be combined with regenerating factors that activate reparative processes in the infarcted heart and pro-survival factors that protect transplanted cells from the aggressive environment of post-MI damaged tissues [5]. Additionally, through their engineered 3D structure, biomaterials permit gas and nutrient transportation and the formation of supportive vascular substructures for blood vessels, maximizing cellular adhesion space and inducing ECM secretion, revascularization, and paracrine processes [44].

As the heart has a contractile nature, multiple mechanical forces act upon cardiac tissues, inducing non-uniform 3D deformations [3]. Thus, biomaterials chosen for cardiac regeneration must be able to withstand forces such as mechanical strain, tensile forces, and shearing forces to fit the natural stretching and compression of the myocardium. Moreover, biomaterial implants should be capable of stimulating electrical conductivity to promote dynamic cardiac tissue functions [45,48].

**Figure 3:**
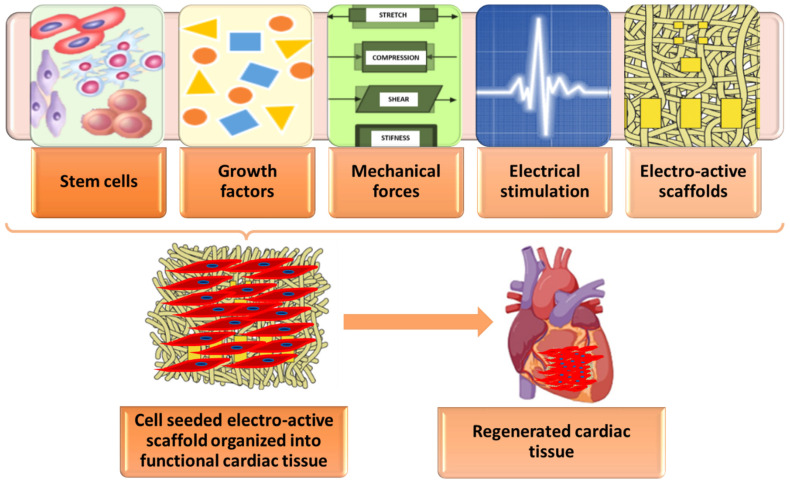
Overview of key factors for cardiac tissue regeneration. Created based on information from literature references [3,4,49].

For clarity, Figure 3 was created to summarize the specific characteristics of biomaterials required for cardiac tissue regeneration.

### 3.2. Approaches for Cardiac Tissue Engineering and Regeneration

Biomaterial-based approaches have gained increasing attention for cardiac tissue engineering and regeneration as recent developments demonstrated promising results in improving cardiac function, promoting angiogenesis, and diminishing adverse immune responses in animal testing and clinical trials [11,50]. To highlight the current progress in cardiac regeneration therapies, the following subsections describe the newest advances in therapeutic delivery via cardiac patches, injectable hydrogels, extracellular vesicles, and scaffolds.

#### 3.2.1. Cardiac Patches

One of the significant focuses of cardiac regeneration approaches is represented by cardiac patches. Such structures can be made from both natural and synthetic materials engineered to have a microstructure that mimics native heart tissues, provides a microenvironment for the incorporated biological moieties, and ensures the necessary support for the construct itself [11,50]. Cardiac patches can impart functional benefits to damaged myocardium, relying on appropriate cell adhesion and proliferation. Fine-tuning cardiac patches according to desired size, shape, and mechanical strength offers enhanced compatibility. Moreover, cardiac patches can be seeded with cells before implantation, allowing their growth and maturation in culture or biological reactors [51].

When placed on the heart surface at the infarction site, these biomaterial structures can improve cardiac function by delivering various bioactive factors or cells [52]. Specifically, scientists have successfully incorporated a wide range of relevant cells, counting synthetic cardiac stromal cells [42], adipose tissue-derived mesenchymal stem cells [53], cardiomyocytes [54,55,56,57,58,59], mesenchymal stromal cells [56], endothelial cells [56], and skeletal myoblast stem cells [60]. Moreover, several studies have also investigated the addition of bioactive molecules, such as hepatocyte growth factor, insulin-like growth factor-1 [61], isoproterenol, and epinephrine [57], to increase the therapeutic efficiency of the patches.

In terms of substrate materials, recent studies mainly approached the use of decellularized porcine biological materials [42,62], natural-based polymers (e.g., cellulose [53,59], chitosan [53,54,63], silk fibroin [53], collagen [55,61], gelatin methacrylate [56]) and synthetic polymers (e.g., polyethylene glycol diacrylate [56], polycaprolactone [60], polyvinyl alcohol [58], polyvinyl pyrrolidone [58]). The advantage of using decellularized natural ECM from native tissues resides in its complex composition of proteins interlaced with proteoglycans that provide the necessary support for cells to orient and interact via signaling factors. Nonetheless, when considering the transition from in vitro and in vivo testing to human studies, these biological materials pose several challenges, including autologous tissue/organ scarcity, host responses, and pathogen transfer when utilizing allogenic and xenogenic tissues [64].

**Figure 4:**
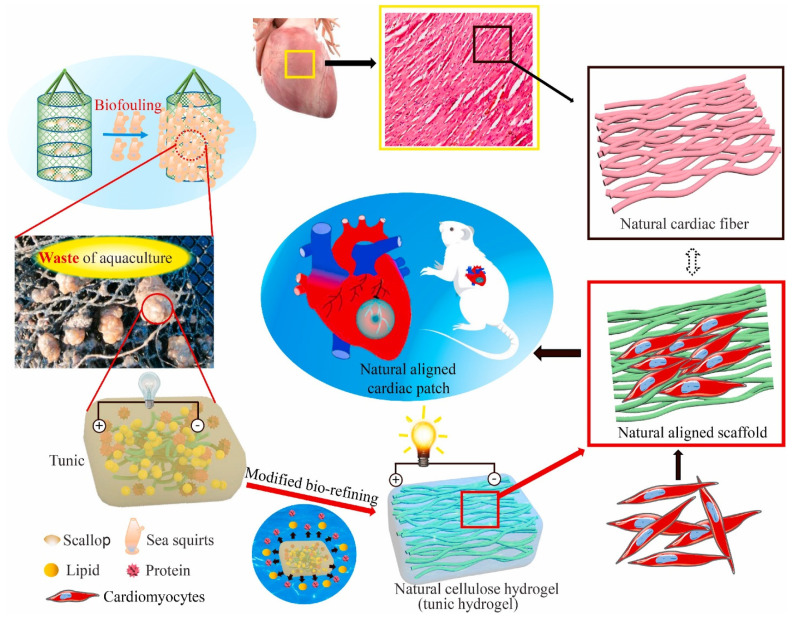
Sea squirts-derived cardiac patch for myocardial infarction from the waste of marine culture. Adapted from [59].

Thus, there is an increased scientific interest in developing cardiac patches from non-animal natural sources or biocompatible synthetic materials. One particularly interesting recent research direction is proposed by He and colleagues [59]. The authors aimed to solve two problems at once by fabricating cardiac patches from waste biomass-sea squirts (Figure 4). The researchers proved that the tunic cellulose-derived natural self-conductive structures successfully served as functional cardiac patches, promoting cardiomyocyte maturation and spontaneous contraction. Therefore, their solution could benefit marine environmental bio-pollution while also upgrading current therapeutic options for MI treatment.

For an at-glance perspective over newly designed cardiac patches, Table 1 presents several recent studies, summarizing information concerning utilized substrate materials, cells, and bioactive molecules, also emphasizing the testing stage and reported observations.

#### 3.2.2. Injectable Hydrogels

Sustained efforts to develop strategies for improving the retention of cell therapy have also resulted in the design and fabrication of various hydrogels that can be injected at the damaged site. The viscoelastic properties of hydrogels contribute to inhibiting applied tensions on the injured region, preventing the formation of fibrous and scar tissue. Moreover, their porosity facilitates stem cell migration to the affected site, positively impacting their retention and survival [43,66,67].

**Figure 5:**
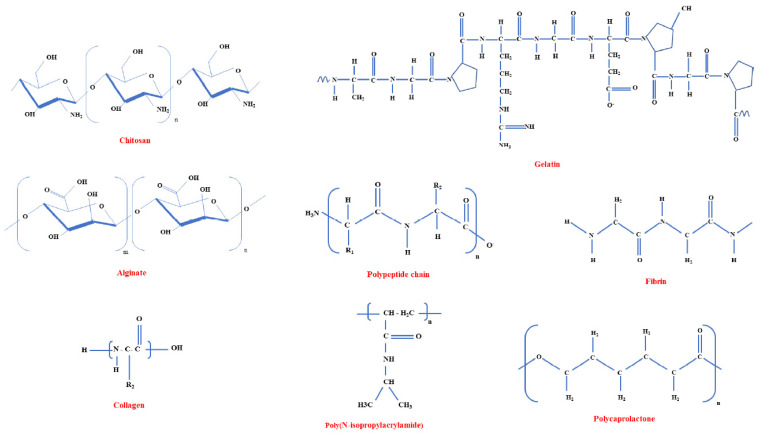
Molecular structures of several injectable hydrogels suitable for cardiac tissue engineering. Adapted from [23].

Hydrogel-type biomaterials can be employed in the delivery of both cellular and acellular biological components. For instance, natural polymers, including alginate, chitosan, collagen, fibrin, fucoidan, hyaluronic acid, and keratin, have been used as efficient injectable carrier materials for active factors [15]. Nonetheless, given their favorable architectures, hydrogels also represent the most commonly utilized cellular scaffold. Specifically, hydrogels form networks due to molecular interactions between the different functional groups present in the base polymer (Figure 5). These structures allow hydrogels to swell upon absorption of biological fluids, serving as soft elastic scaffolds resembling native tissue microenvironments [23,68].

In what concerns its working principle, hydrogel material in its liquid form can be mixed with cells and biomolecules of interest and injected as a solution to the target myocardial area, where it undergoes gelation, becoming a mixed 3D cell polymeric network ready for integration with the surrounding tissue. Thus, injectable hydrogels offer a minimally invasive therapeutic alternative for creating the protective environment cells need to survive and bioactive agents to exert their intended activity [1,5,11,69,70]. Additionally, the high-water content of hydrogels endows them with the ability to efficiently exchange nutrients and metabolic waste products with the surrounding environment. Moreover, hydrogel formulation can be fine-tuned to mimic the mechanical properties of the native ECM and provide cells with the biochemical stimuli required for directing them toward desired fates [66].

Depending on the nature of the base material, hydrogels can be divided into natural and synthetic. Natural hydrogels made of polysaccharides or proteins are considered appealing due to their non-toxicity, immunogenicity, and excretion of metabolites. Moreover, superior water-swelling properties allow them to easily adsorb and contain nutrients and small molecules, upgrading cell survival and boosting exercise performance. In contrast, synthetic hydrogels are recognized for their stronger mechanical properties and the feasibility of physically or chemically linking to new functional groups to enhance functionality. Synthetic hydrogels also benefit from a low risk of immune rejection but present low adhesion and not as good biocompatibility as that of natural materials [70].

One particularly appealing cardiac repair strategy is the use of self-healing injectable hydrogels that mechanically support infarcted tissue and prevent pathological ventricular remodeling while repairing their own structure and regaining original properties following damage [71,72,73]. Self-healing hydrogels can be functionalized with diverse growth factors, pro-angiogenic cytokines, microRNAs (miRNAs), and stem or progenitor cells to enhance cardiac tissue regeneration [74]. Specifically, studies have shown that stem cell-laden hydrogels lead to better effects than either stem cell transplantation or hydrogel injection alone, achieving synergistic potential in recovering infarcted myocardium [43,75]. Moreover, enriching hydrogels with antioxidative properties can lower the oxidative stress levels from ischemic myocardium, whereas providing immunomodulatory activity contributes to decreasing postinfarct inflammatory response [74].

Given the tremendous potential of injectable hydrogels, researchers have investigated various formulations for cardiac repair and regeneration. For instance, Traverse Jay et al. [76] have investigated the safety and feasibility of VentriGel in early and late post-MI patients with left ventricular dysfunction. The decellularized ECM hydrogel was injected transendocardially into 15 patients as the first-in-man study concerning this material. In terms of efficacy, the study revealed improvements mainly in patients who had MI more than 12 months before treatment.

Alternatively, Contessotto et al. [77] proposed the use of an ECM-mimicking hydrogel that can be intramyocardially injected. Tested on an ovine model, the injectable hydrogel made of elastin-like recombinamers led to complete functional recovery of ejection fraction 21 days after the intervention. Moreover, fibrosis was diminished, angiogenesis was stimulated, and GATA4+ cardiomyocytes were better preserved in the border zone of the infarct.

In contrast, Bai et al. [78] have created an injectable temperature-sensitive hydrogel based on ECM from decellularized rat hearts seeded with cardiomyogenic cells isolated from brown adipose. The synergistic combination led to the preservation of cardiac function and chamber geometry, as the ECM hydrogel enhanced cell engraftment and myocardial regeneration.

Following a different approach, Xu and colleagues [79] have developed biodegradable hybrid hydrogels based on thiolated collagen and multiple acrylate-containing copolymers. Alone or encapsulated with bone marrow mesenchymal stem cells, these hydrogels were able to increase ejection fraction and improve cardiac function at 28 days after administration into rat MI model. Anatomically, the injected formulation considerably diminished the infarct size and increased the wall thickness.

Dong et al. [80] have fabricated a self-healable conductive hydrogel based on chitosan-graft-aniline tetramer and dibenzaldehyde-terminated poly(ethylene glycol) that can be injected into infarcted hearts. The injectable hydrogels present good biocompatibility, biodegradability, tunable release rate, and a conductivity of ∼10^–3^ S·cm^–1^, which is similar to native cardiac tissue.

Another self-healing hydrogel was proposed by Hu et al. [81], who used recombinant humanized collagen type III for the delivery of curcumin nanoparticles at the MI site. The natural drug exhibited remarkable antioxidant and anti-inflammatory activity, effectively reducing ROS levels, cell apoptosis, and post-MI inflammatory reactions, while the biomaterial stimulated cell proliferation, migration, and angiogenesis. The synergistic approach led to rapid recovery of cardiac function, rendering this multifunctional cytocompatible hydrogel a promising tool for regenerating infarcted hearts.

Navaei and colleagues [82] have manufactured a mechanically robust injectable hydrogel made of gelatin crosslinked to temperature-responsive poly(N-isopropylacrylamide) for cardiac cells delivery and tissue engineering. The hybrid material offered bioactivity and enhanced water content, resulting in optimum cell survival, adhesion, and spreading, also leading to cytoskeletal and cardiac-specific markers organization. The injectable matrix was able to accommodate cardiac fibroblasts, augmenting the functionality of the cell-embedded hydrogel.

On a different note, Waters et al. [83] have prepared a gelatin and Laponite^®^-based injectable hydrogel to deliver therapeutic biomolecules (secretome) secreted by human adipose-derived stem cells. The biocompatible system significantly increased capillary density, reduced scar area, and improved cardiac function, demonstrating its potential as an MI treatment.

#### 3.2.3. Extracellular Vesicles

**Figure 6:**
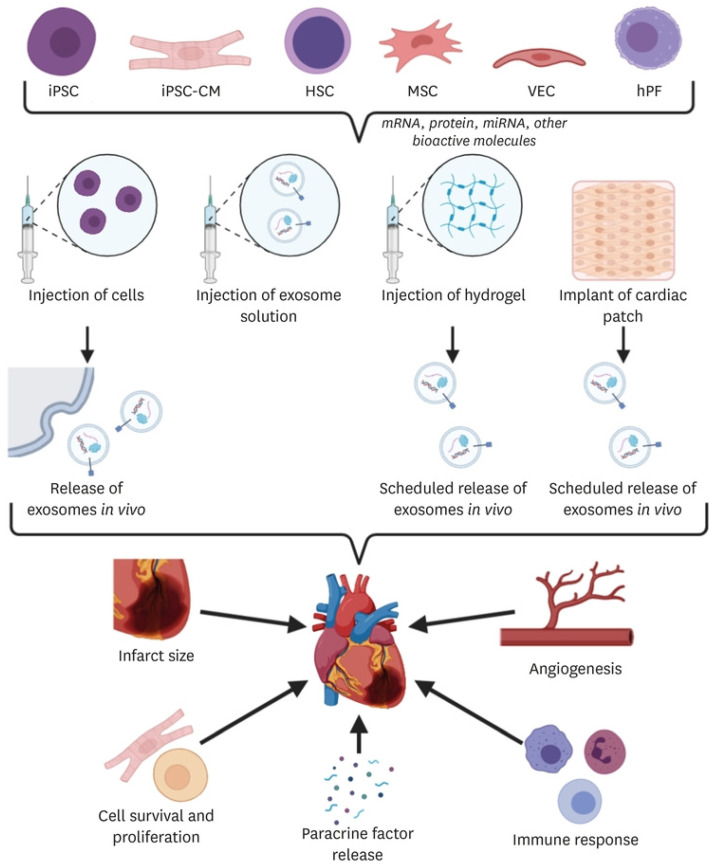
Schematic representation of possible strategies involving extracellular vesicles for endogenous cardiac regeneration. Adapted from [11].

Besides the above-discussed cardiac repair techniques, extracellular vesicles in general and exosomes in particular hold great promise for designing performant alternative or complementary formulations for cell therapies, cardiac patches, and injectable hydrogels (Figure 6). Their potential resides in the role played in regulating cardiac function, as it was recently discovered that extracellular vesicle dysregulation might be an important mechanism of injury progression [2,84,85]. Moreover, exosome content is beneficial for cardiac regeneration as the combination of messenger RNAs (mRNAs), proteins, miRNAs, and other bioactive molecules facilitates angiogenesis, reduces infarct size, ensures cell survival and proliferation, releases paracrine factors, and modulates immune response [11,84,86,87].

Extracellular vesicles originating from various cells have demonstrated potential for cardiac regeneration therapy either alone or in different combination approaches [85]. The use of hydrogels or cardiac patches as delivery vehicles allows tailored release of extracellular vesicles, whereas the injection of parent cells results in a variable release [11]. Thus, most of the recent studies have approached extracellular vesicle-based treatments in association with various biomaterials as convenient biocompatible carriers for creating multifunctional therapeutic platforms.

For instance, Lv et al. [88] tackled the potential of mesenchymal stem cell (MSC)-derived small extracellular vesicles for treating MI. In this respect, the authors have incorporated them into alginate hydrogel to improve their retention in the cardiac tissue and enhance the therapeutic outcomes. The as-designed treatment was reported to considerably decrease cardiac cell apoptosis, promote macrophages polarization at day 3 after MI, and increase scar thickness and angiogenesis at 4 weeks post-infarction, thus leading to overall improved cardiac function and infarct size. Similarly, Shao et al. [89] demonstrated the cardiac repair potential of MSC-derived exosomes, comparing their effects to MSCs. Their study concluded that the exosome-based treatment stimulated cardiomyocyte proliferation, inhibited H_2_O_2_-induced apoptosis, and hindered TGF-β induced transformation of fibroblast cells into myofibroblast, leading to better therapeutic activity than MSCs.

Exosomes derived from human umbilical cord MSCs can also be employed in cardiac regeneration, as demonstrated by Han et al. [90]. The authors encapsulated these exosomes in functional peptide hydrogels to improve their retention and stability. In comparison to exosome treatment alone, the hybrid formulation was noticed to bring better results in terms of reducing inflammation, fibrosis, and apoptosis, and promoting angiogenesis, overall leading to an improvement in myocardial function.

Alternatively, Chen and colleagues [91] harvested extracellular vesicles from endothelial progenitor cells (EPCs), incorporating them into an injectable shear-thinning gel. The novel formulation was observed to enhance peri-infarct vascular proliferation, allow preservation of ventricular geometry and positively impact the hemodynamic function post-MI.

Differently, Liu et al. [92] have chosen to work with extracellular vesicles secreted from cardiomyocytes derived from induced pluripotent stem cells. When encapsulated into an engineered hydrogel patch, these vesicles were able to reduce arrhythmic burden, promote ejection fraction recovery, decline cell apoptosis 24 h post-MI, and diminish infarct size and cell hypertrophy 4 weeks post-MI.

#### 3.2.4. Scaffolds

Interesting possibilities have also been envisaged by creating myocardial tissue grafts based on preformed implantable scaffolds. Cardiac tissue engineering scaffolds can be fabricated via crosslinking of biomaterial solution into the desired shape, followed by solidification and/or drying/freeze-drying to generate a porous ECM-like matrix. Compared to hydrogels, scaffold preparation allows more control over the porosity of the structure before cell seeding, also significantly reducing cell exposure to stress during mixing and molding procedures [5]. Moreover, the involvement of advanced manufacturing techniques such as electrospinning, self-assembled monolayers, 3D bioprinting, and thermally induced phase separation allows the combination of the substrate material with peptides and DNA for creating biomimetic 3D scaffolds able to support the regeneration of various stem cells down multiple lineages [44,93]. Additionally, through their customized porous architecture, scaffolds can influence cardiac cell alignment to organize into the gross conformation of native cardiac tissue [5].

Several factors have been observed to be particularly relevant for cardiac applications, counting substrate geometry, stiffness, matrix topography, and electrical stimulation. These aspects must be thoroughly tailored to ensure the differentiation of seeded cells toward achieving high specific functionality as they are known to significantly influence cellular behavior, regulation of motility, proliferation, and differentiation responses [44,45,93]. In this respect, several studies have investigated various scaffolds based on different substrate materials, embedded nanostructures, and seeded cells, a few examples being described in more detail below.

Hayoun-Neeman et al. [94] have created macroporous scaffolds based on alginate in either pristine form or modified with arginine-glycine-aspartate (RGD) peptide and heparin-binding peptide (HBP). The scaffolds were seeded with human ESC-derived cardiomyocytes and human dermal fibroblasts to form functional cardiac tissues. Alginate modification with peptides was noticed to improve the biomaterial’s functionality. Its potential for cardiac regeneration was demonstrated by an increase in contraction amplitude and calcium transients with time, a decrease in excitation threshold, and a display of typical fiber morphology with massive striation.

Tamimi et al. [10] have constructed ternary scaffolds made of solubilized ECM, chitosan, and alginate in different blending ratios. All samples had porosities exceeding 96% and very high swelling rates while maintaining their stability in PBS solution. Moreover, the addition of polysaccharides was noted to improve the tensile strength of the designed scaffolds. Nonetheless, the best results were obtained for the mixture containing 75% ECM, 12.5% alginate, and 12.5% chitosan, which improved human MSCs proliferation and produced a higher cardiac marker expression.

Liang et al. [95] have recently developed a conductive scaffold using polypyrrole blended with silk fibroin solutions. The researchers tested various silk fibroin concentrations and different polymer blending ratios, obtaining the closest mechanical properties to the native heart tissues for 7% silk fibroin solution and sufficient electrical conductivity for cardiomyocytes with a polypyrrole-to-silk fibroin ratio of 15:85.

Alternatively, Li and colleagues [96] have incorporated high-aspect-ratio gold nanowires into gelatin methacrylate (GelMA) hydrogels to obtain biomaterial scaffolds with enhanced electrical conductivity and mechanical properties. Their scaffold provided a proper medium for constructing functional cardiac tissue as cardiomyocytes cultured on it demonstrated better cell viability and maturation state than those cultured on plain GelMA hydrogels, also displaying synchronous beating activity and a faster spontaneous beating rate on nanocomposite hydrogels.

Saravanan et al. [97] have also utilized gold as a component for designing an advanced scaffold for cardiac repair. The authors have created a conductive biodegradable structure by incorporating graphene oxide gold nanosheets into a chitosan matrix. The synthesized scaffold presented well-controlled porous architecture, swelling, and degradation properties, supporting cell attachment and growth without signs of cytotoxicity. Following implantation, the scaffold improved cardiac contractility and restored ventricular function.

**Figure 7:**
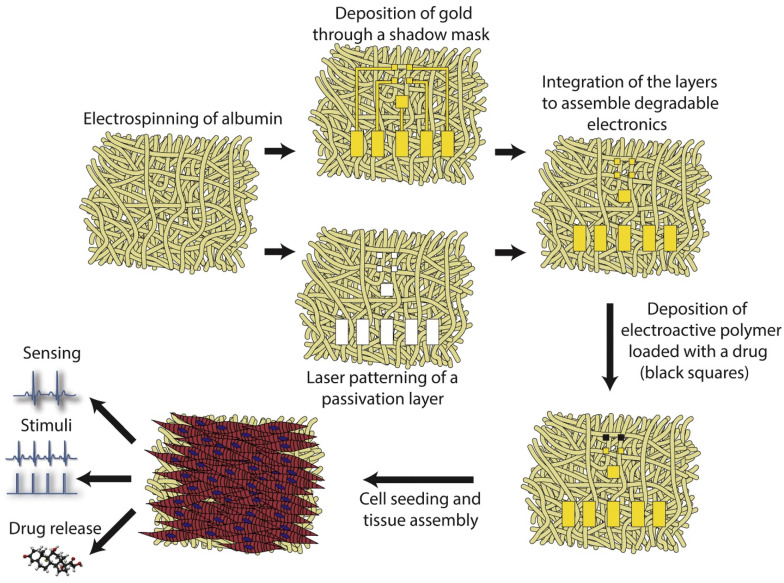
Schematic representation of the advanced scaffold developed by Feiner et al. Electrospun albumin creates two thick mats: the first is covered with shadow masks and 600 nm thick Au electrodes are evaporated onto it, and the second mat is used as a passivation layer. The layers are stuck together using an ECM-based hydrogel as an adhesive. Next, a layer of polypyrrole loaded with dexamethasone is deposited onto selected electrodes. Neonatal rat ventricular cardiomyocytes are seeded onto the device that permits further organization into functional cardiac tissue. With the aid of an external amplifier, extracellular signals may be recorded from the tissue, and stimuli may be delivered to the device for pacing and drug release. Adapted from [49].

On a different note, Feiner and colleagues [49] have fabricated an elastic biodegradable electronic made of electrospun albumin fibers serving as substrate and passivation layer for evaporated gold electrodes (Figure 7). The performant scaffold allowed cardiomyocytes to organize into functional cardiac tissue, enabled actuation of the engineered tissue, and triggered drug release. Moreover, the electronic scaffolds degraded post-implantation, which makes them an appealing candidate for short-term in vivo application.

## 4. Summative Discussion and Future Perspectives

To address the limitations of current cardiac injury treatments, extensive research has been noted in developing biomaterial-based strategies. Special focus was observed on using various natural and synthetic polymeric materials to create delivery platforms for different cells and bioactive molecules relevant to cardiac tissue engineering and regeneration. Recent research efforts have materialized into a number of cardiac patches, injectable hydrogels, extracellular vesicle-based therapies, and advanced scaffolds that showed promising results when tested in vitro and in vivo. Nonetheless, the proposed innovative treatment strategies have not yet reached the stage of clinical testing, except for a few studies (Table 2). Specifically, scientists have translated pre-clinical research to human testing of several biomaterial approaches, including VentriGel [98], CorMatrix-ECM [99], PeriCord [100], and epicardial atrial appendage micrograft (AAM) patch [101]. 

Most of the tabulated studies do not have publicly posted results, with the exception of NCT02887768 [99], whose pre-clinical observations have been debated in several publications [102,103]. It has been reported that the use of bioinductive ECM biomaterial (i.e., CorMatrix Cardiovascular Inc, Roswell, Ga) promotes endogenous myocardial repair and functional recovery after MI, its clinical translation being considered a promising adjuvant therapy to surgical revascularization.

Moreover, undergoing clinical studies may soon confirm the utility of novel biomaterials for cardiac tissue engineering and regeneration, leading to the entrance into the clinical practice of performant treatment alternatives. In addition, investigating the effectiveness and safety of the discussed biomaterial formulations in humans represents a mandatory step in introducing improved therapeutic approaches in the clinical setting.

Another appealing future research direction consists in producing multi-material structures via 3D printing for accurately recapitulating natural cardiac tissues and customizing the implanted device to particular patient needs [15,104,105,106,107]. However, although cardiovascular structures such as vasculature constructs, heart valves, and myocardium have been successfully 3D bioprinted, these techniques are still in their infancy, requiring further optimization studies. Specifically, in-depth research is needed to accurately construct cardiac analogs with full functionality and complex micro-architecture [108].

One more perspective for creating personalized treatment approaches is machine learning. These advanced computational models are expected to play greater and greater roles in tissue culture, biomaterial development and fabrication, animal models, and clinical research [109]. In combination with high-throughput theoretical predictions and high-throughput experiments, machine learning represents a shifting paradigm from conventional trial and error studies, leading to a faster technological advancement in materials fabrication [110]. Specifically, artificial intelligence algorithms have the potential to improve the design and processing of micro-physiological systems and help in the optimization stages toward maximizing survival rates [44,106,111,112]. Moreover, the synergic use of medical imaging and modern computational algorithms can improve myocardial textural analysis toward identifying new biomarkers, thus addressing the need for novel clinical endpoints. Additionally, based on phenogrouping through radiomics signatures, machine learning algorithms would also enable appropriating patients likely to respond to stem cell therapy [113], allowing for customized treatments of maximum efficacy.

Nanotechnology also offers good prospects for tissue engineering and regeneration [114,115,116,117,118,119]. More specifically, nanomaterials can be integrated into the composition of advanced cardiac-mimicking architectures to enhance the functionality and physicochemical properties of the composite constructs [120]. The association of biomaterials with nanoparticles [55,81,86,121,122], nanofibers [53,60,123,124], nanowires [96,125], and nanosheets [97,126,127] can positively influence cardiac repair. Moreover, nanostructures can be used as carriers for the targeted delivery of therapeutic agents of interest for cardiovascular disease treatment [128,129,130].

The emerging technology of tissue-on-a-chip platforms also holds great promise for finding better treatments for cardiovascular diseases. Microfluidic chips allow the investigation of human physiology in a controlled, isolated, and accessible environment, being suitable devices for relevant disease models and drug screening systems [2,7,131,132,133,134,135,136]. Newly developed advanced microfluidic platforms [137] are expected to clarify cellular and molecular mechanisms specific to relevant cardiovascular diseases and unveil the response of damaged cardiac tissues to various tested therapeutic strategies [136,137,138,139,140].

## 5. Conclusions

To conclude, tailoring biomaterials to meet the complex set of requirements imposed by cardiac tissue engineering and regeneration is gaining increasing attention from the scientific community. The ingenious combinatorial use of natural and synthetic polymers, electrically conductive materials, stem and/or progenitor cells, and bioactive molecules can revolutionize the manner cardiac injuries are managed. Embracing an interdisciplinary approach, a recently developed series of cardiac patches, injectable hydrogels, extracellular vesicle-based formulations, and biomaterial scaffolds have already been demonstrated effective when tested in vitro and in vivo. However, there is still room for improvement to optimize the proposed approaches and deepen investigations to ensure technology transfer to the clinical setting.

## Figures and Tables

**Table 1:** Examples of recently developed cardiac patches.

Substrate	Cells	Bioactive Molecules	Testing	Observations	Ref.
Decellularized porcine myocardial extracellular matrix	Synthetic cardiac stromal cells	-	Rat and porcine models of acute MI	Supports cardiac recovery Reduces scarring Promotes angiomyogenesis Boosts cardiac function The patch is clinically feasible and easy to store	[42]
Decellularized porcine myocardium slice	-	-	Rat model of acute MI	Firm attachment to host myocardium Prevents thinning of the left ventricular wall Allows infiltration of a large number of host cells Significant improvement of left ventricle wall contraction and cardiac functional parameters	[62]
Cellulose nanofibers modified with chitosan/silk fibroin (CS/SF) multilayers	Adipose tissue-derived mesenchymal stem cells	-	Rat model of acute MI	Less ventricular remodeling than direct cell injection Elevates left ventricular ejection fraction and fractional shortening Attenuates cardiac fibrosis and apoptosis Promotes local neovascularization	[53]
Chitosan films micropatterned with a re-entrant honeycomb (bowtie) pattern and coated with polyaniline and phytic acid	Neonatal rat ventricular myocytes and fibroblasts	-	Rat MI model	Conductive and cytocompatible patch No detrimental effect on the electrophysiology of both healthy and MI hearts Conform better to native heart movements than unpatterned patches No detrimental effect on cardiac function Negligible fibrotic response after two weeks	[54]
Collagen-based hybrid nanocomposite loaded with nanogold	Neonatal rat cardiomyocytes	-	Murine model 7 days post-MI	Increases connexin-43 expression in cells cultured under electrical stimulation Able to recover cardiac function Increased blood vessel density Reduces scar formation	[55]
Collagen patch incorporated with alginate microparticles	-	Hepatocyte growth factor Insulin-like growth factor-1	Isolated myocardial tissue from rats	Extends the release of encapsulated proteins up to 15 days Increases motogenic and proliferative effect Favors the natural regenerative potential of cardiac stem cells	[61]
Gelatin methacrylate and polyethylene glycol diacrylate-based patch with myocardial fiber orientation	Cardiomyocytes, mesenchymal stromal cells, and endothelial cells	-	Mice model of chronic MI with ischemia-reperfusion	Increases cell density Reduces damaged tissue area Ensures high engraftment rates Strong integration within the epicardium Progressive implant vascularization	[56]
Fibrin gel-based 3D patch	Cardiac myocytes reprogrammed from human adipogenic mesenchymal stem cells	Isoproterenol Epinephrine	In vitro	Increases the expression of mTOR, KCNV_1_, GJA_5_, KCNJ_16_, CTNNT_2_, KCNV_2_, MYO_3_, FOXO_1_ and KCND_2_ Restores the electrical activity of infarcted hearts Improves cardiac functions	[57]
Polycaprolactone nanoscale-to-microscale fibers	Skeletal myoblast stem cells	-	Rat MI model	Presents strong compliance and survival after transplantation Release VEGF	[60]
Polyvinyl alcohol and polyvinyl pyrrolidone-based patch	Neonatal mouse cardiomyocytes	-	Rat model	Biocompatible and biodegradable No signs of tissue damage or necrosis at the implantation site, no detectable wound complications, inflammatory response, or adverse tissue reactions	[58]
Fibrin gel-based patch with microengineered blood vessels	Human umbilical vein endothelial cells and human cardiac stem cells	-	Rat model of acute MI	Induces profound mitotic activities of cardiomyocytes Significantly enhances myocardial capillary density	[65]
Porous self-conductive cellulose hydrogel	Cardiomyocytes	-	Rat model of acute MI	Significantly promotes the maturation and spontaneous contraction of cardiomyocytes Enhances cardiac function of animal models	[59]

**Table 2:** Clinical trials involving biomaterials for cardiac tissue engineering and regeneration.

ClinicalTrials.gov Identifier	Official Title	Intervention/ Treatment	Phase	Reference
NCT02305602	A Phase I, Open-label Study of the Effects of Percutaneous Administration of an Extracellular Matrix Hydrogel, VentriGel, Following Myocardial Infarction	Biological: VentriGel	Phase 1	[98]
NCT02887768	Cardiac Infarct Repair Using CorMatrix^®^-ECM: Clinical Feasibility Study	Device: Epicardial Infarct Repair with CorMatrix-ECM Procedure: Coronary Artery Bypass Grafting Surgery	Early Phase 1	[99]
NCT03798353	Pericardial Matrix with Mesenchymal Stem Cells for the Treatment of Patients With Infarcted Myocardial Tissue (The PERISCOPE Trial)	Combination Product: PeriCord: Expanded and cryopreserved allogeneic umbilical cord Wharton’s jelly-derived adult mesenchymal stem cells colonized on human pericardial matrix. Procedure: Surgery by sternotomy	Phase 1	[100]
NCT05632432	Atrial Appendage Micrograft Transplantation in Conjunction with Cardiac Surgery—the AAMS2 Randomized Controlled Trial	Procedure: Epicardial AAMs-patch transplantation Diagnostic Test: RNA-stabilized whole blood sampling Diagnostic Test: Plasma sampling Diagnostic Test: Transthoracic echocardiography Diagnostic Test: Late-gadolinium enhancement cardiac magnetic resonance imaging (LGE-CMRI) Other: Symptom-scaling Other: 6 min walking test (6MWT) Diagnostic Test: Blood sampling (NT-proBNP) Diagnostic Test: Transesophageal echocardiography	Not applicable	[101]

## Data Availability

Not applicable.
